# Elevated serum expression of p53 and association of TP53 codon 72 polymorphisms with risk of cervical cancer in Bangladeshi women

**DOI:** 10.1371/journal.pone.0261984

**Published:** 2021-12-28

**Authors:** Md Shaki Mostaid, Sadia Biswas Mumu, Md Aminul Haque, Shahana Sharmin, Mohd Raeed Jamiruddin, Ghazi Muhammad Sayedur Rahman, Hasan Mahmud Reza

**Affiliations:** 1 Department of Pharmaceutical Sciences, School of Health and Life Sciences, North South University, Dhaka, Bangladesh; 2 Department of Pharmacy, Brac University, Dhaka, Bangladesh; Tanta University Faculty of Medicine, EGYPT

## Abstract

Differential expression of p53 has been reported in cervical cancer, primarily in tumor tissue biopsies. In this study, we examined the association of TP53 codon 47 and codon 72 polymorphisms and serum level expression of p53 in cervical cancer patients (n = 129) and healthy controls (n = 122). We found elevated levels of serum p53 protein levels in cervical cancer patients (p = 0.0442) compared to healthy controls. Moreover, we found higher levels of serum p53 in patients with grade-III tumor (p = 0.001) compared to healthy controls. Examination of SNPs showed TP53 Arg/Pro heterozygosity (adjusted OR = 2.126, 95% CI = 1.181–3.827, p = 0.012), Pro/Pro mutant homozygosity (adjusted OR = 3.564, 95% CI = 1.647–7.713, p = 0.001), along with the combined genotype (Arg/Pro+Pro/Pro) (adjusted OR 2.542, 95% CI = 1.517–4.260, p<0.001) significantly increases the risk of cervical cancer. Expression quantitative trait analysis revealed no significant association with protein expression. Our results represent for the first time the upregulation of serum p53 in cervical cancer in Bangladeshi women and supports the association of TP53 codon 72 polymorphisms with cervical cancer.

## Introduction

Cervical cancer is one of the most commonly reported gynecological malignancies. It ranks fourth as the most frequently diagnosed cancers and the fourth leading cause of cancer-related death in females worldwide [1]. In Bangladesh, it accounts for 12% of all newly diagnosed cancer cases in females with a mortality rate of 4.6% [2]. Although human papillomavirus is considered as one of the prime factors for the development of cervical cancer [3–5], studies have reported the involvement of several other genetic and environmental risk factors in cancer development and prognosis [6–8].

Several studies have reported an association between genetic polymorphism and susceptibility to cervical cancer in different ethnic populations [9,10]. TP53 is a tumor suppressor gene that plays a vital role in apoptosis, DNA repair mechanism, regulation of cell cycle, and suppressing angiogenesis [11,12]. Single nucleotide polymorphisms (SNPs) of the TP 53 gene, especially codon 47 (rs1800371) and codon 72 (rs1042522) have been heavily linked to different types of malignancies including cervical cancer, but results have been inconsistent [13–18]. The CC mutant genotype that codes for the proline allele at codon 72 has been associated with an increased risk of cervical cancer in Bengali women from the Indian subcontinent [13,19]. This finding is also supported by studies with cervical cancer patients from Han Chinese and Latin American ethnicity [17,18,20]. These findings have been collaborated with the meta-analysis study which reported that Pro/Pro genotype and proline allele was associated with increased risk of cervical cancer within the Indian population [21]. On the contrary, the proline allele was found to confer a protective effect in patients with cervical cancer from Saudi Arabia [22]. The proline allele has also been linked with increased overall survival in Caucasian cervical cancer patients [23]. On the other hand, the normal homozygous GG genotype, that codes for the arginine allele, has been associated with increased risk of cervical cancer in Serbian and Kyrgyz women [24,25]. Reports of no statistically significant association between TP53 codon 72 polymorphisms with cervical cancer are also available [26,27]. As a result, the exact role of TP53 codon 72 polymorphisms in cervical cancer remains largely unknown.

P53 is a highly complex protein which has been found to be involved in cancer progression, neurodegenerative diseases, and ageing [28]. Several isoforms of p53 exists that are detectable in wild type as well as in truncated form and most of the N-terminal truncated isoforms are suggested to be involved in carcinogenesis [29]. However, protein level detection of all the isoforms is complex due to the lack of structural and functional characterization. Serum p53 is a promising biomarker and several studies have been conducted on the expression level of p53 in different types of malignancies including cervical cancer [30–33]. Elevated levels of mutant serum p53 have been reported in patients with cervical cancer [30,34,35]. Evaluation of serum p53 as a biomarker in other types of cancers produced inconsistent results [31–33]. A recent study in the North-East Indian population reported downregulation of p53 protein expression in tissue biopsies of cervical cancer patients compared to the non-neoplastic adjacent control region. Moreover, they reported that p53 protein was downregulated in lower grades (≤ stage III) of cervical cancer compared to higher grades (≥ stage III) [36]. In contrast, p53 expression was upregulated in stage IB1 cervical carcinoma in Taiwanese cervical cancer patients [37]. No study has been conducted on the serum expression level of p53 in Bangladeshi cervical cancer patients.

The polymorphism at codon 47 is caused by a substitution of the proline with serine residue that results in a significant decrease in the tumor suppressor function of the p53 protein. Two previous case-control studies found no significant association of this polymorphism with lung cancer and cervical cancer in the Bangladeshi population [13,14]. No studies looked at the potential cis-regulatory effect of this SNP on serum p53 protein expression.

The purpose of this study was to evaluate the association of TP53 codon 72 and codon 47 polymorphisms with cervical cancer risk in the Bangladeshi population. Furthermore, we looked into the association of the SNPs with clinicopathological characteristics. We investigated the serum expression level of p53 in cervical cancer and identified the differences in expression in cases with different histopathological characteristics. Finally, we investigated if codon 47 and codon 72 have any cis-regulatory effect on serum p53 protein expression.

## Materials and methods

### Selection of study population

The study comprised 129 cervical cancer patients and 122 age-matched healthy controls. Cervical patients were recruited from two hospitals named Dhaka Medical College and Hospital, Dhaka and Ahsania Mission Cancer and General Hospital, Dhaka, Bangladesh in between November 2019 and April 2021. Required sample size was calculated a priori using G*power software with an effect size, d = 0.8 (large effect) and α = 0.05 which yielded in a total required sample size of 252. The patients were histologically diagnosed with cervical cancer by expert oncologists and classified according to the International Federation of Gynecology and Obstetrics (FIGO) staging criteria [38]. All the patients were practicing teetotalism throughout their life. The age-matched healthy controls were selected after thorough medical examination and those with a history of mental illness, head injury, trauma, pregnancy, substance abuse, alcohol consumption was excluded from the study. A written consent form was taken from all study participants and the study was conducted according to the declaration of Helsinki and its further amendments [39]. The study protocol was approved by the ethical review committee of the Dhaka Medical College and Hospital (DMCH), Dhaka. Laboratory experiments were conducted at the Department of Biochemistry and Molecular Biology, University of Dhaka; Department of Pharmaceutical Sciences, North South University, Dhaka and Rufaida Research Foundation, Dhaka.

### DNA extraction and genotyping

10 ml of blood was collected from each study participant. 3 ml was collected in potassium EDTA tubes (BD Vacutainer^®^ blood collection tubes, Becton and Dickinson and Company, NJ, USA) for genotyping. 7 ml blood was collected in BD Vacutainer^®^ serum separator tubes for collection of serum. All samples were stored at -80°C until further experiments. DNA extraction was performed using Bioneer^®^ genomic DNA extraction kit (Bioneer Corporation, Daejeon, South Korea) using the manufacturer’s protocol. The quality of DNA was examined using NanoDrop spectrophotometer (ThermoFisher Scientific, Waltham, MA, USA). The selected SNPs were genotyped using the Polymerase chain reaction-restriction fragment length polymorphism (PCR-RFLP) using the previously described method [14]. The PCR products of TP53 codon 72 (309 bp) and TP53 codon 47 (201 or 185 bp) were digested for 16 hours with restriction enzymes *BstUI* and *MspI* respectively. Next, the amplicons were visualized on 2% agarose gel using ethidium bromide by gel electrophoresis. Table 1 contains a summary and S1 Table contains the details of the PCR protocol.

**Table 1:** Primers for *TP53* codon 47 and codon 72.

Target codon	Sequence	PCR product (bp)	T_m_(°C)
*TP53* codon 47 (rs1800371)	F 5′-CTG GTA AGG ACA AGG GTT GG-3′ R 5′-TCA TCT GGA CCT GGG TCT TC-3′	201 or 185^a^	62
*TP53* codon 72 (rs1042522)	F 5′-TTC ACC CAT CTA CAG TCC-3′ R 5′-CTC AGG GCA ACT GAC CGT-3′	309	60

^a^ Size divergence due to a 16-bp in/del intronic polymorphism near the *TP53* Pro47Ser single nucleotide polymorphism.

### Protein quantification

TP53 ELISA Pair Set (catalog number: SEK90001, Sino Biological, Beijing, China) was used to measure p53 levels in serum according to the manufacturer’s protocol. This ELISA assay detects the full length p53 protein covering the oligomerization domain, transcriptional activation domain and DNA binding domain. At first, the capture antibody was brought to a working concentration (2μg/ml) and each well of the 96-well microplate was coated with 100μl of the diluted antibody, sealed, and incubated overnight at 4°C. After that, each well was aspirated and washed with 300μl of wash buffer three times and blotted against a clean paper towel. Next, 300μl of blocking buffer was added to each well and incubated at room temperature for one hour. Aspiration and washing were performed for the second time to prepare the plates for sample addition.

25μl of serum was diluted to 100μl with sample dilution buffer for the assay. 100μl of serum or standards were added in duplicate to wells of the microplate, sealed, and incubated at room temperature for 2 hours. Then the plates were washed three times with wash buffer. After that, 100μl of the detection antibody was added to each well and incubated at room temperature for 1 hour. After washing the plates three times with wash buffer, 200 μl of substrate solution was added to each well and incubated for 30 minutes in dark at room temperature. Next, 50μl of stop solution was added and absorbance was measured at 450nm using a microplate reader.

A standard curve was generated for each assay by plotting the mean absorbance for each standard concentration vs. the corresponding p53 concentration. The standard curve (r^2^ ≥ 0.99) was generated with a four-parameter logistic curve fit. The concentration of p53 in the serum samples was obtained by interpolating the absorbance values using the standard curve in GraphPad Prism 9. The final concentrations were obtained by multiplying with the dilution factor (x4).

### Statistical analysis

Two-tailed tests were used for all statistical analyses. Independent sample t-test was used for the continuous variables and chi-square test was used for categorical variables to compare the demographic data, clinicopathological data, and genotypes frequencies between cases and controls. The Chi-square test was also used to measure the deviation of the genotype frequencies in the control group from the case group to calculate the Hardy-Weinberg Equilibrium (HWE). Multivariate logistic regression was used to measure the adjusted odds ratio with 95% confidence intervals and P values.

### Protein expression analysis

Q-Q plots and Shapiro-Wilk test were used to test the normality of distribution of serum p53 protein expression in cases and controls (S1 Fig). As the protein expression data were not normally distributed, generalized linear model was used to compare the serum p53 protein expression between cases and controls adjusting for age, parity, contraception, menstruation status. Kruskal-Wallis test was used to measure the difference in p53 protein expression between controls and cases with different histopathological characteristics (stage of cancer, tumor grade, type of cancer). Post-hoc multiple comparisons between groups were performed by Dunn’s multiple comparison test to get the adjusted P values.

### Expression quantitative trait loci analysis

General linear models (GLMs) were used to find the cis-regulatory effects of the SNPs on serum p53 expression. Each model included genotype, case status, and genotype x case-status as covariates. Significant genotype x case status interactions were analyzed post-hoc by case status stratification analysis.

P<0.05 was regarded as significant. The statistical analyses were performed by IBM SPSS statistics, version 23. Graphs were prepared by GraphPad Prism 9.

## Results

### Characteristics of the study population

A total of 251 participants were included in this study where 129 were cervical cancer patients and 122 were healthy controls. Table 2 contains the details of the demographic variables and clinicopathological characteristics. 45 years was taken as a cut-off to separate early and delayed diagnosis of cervical cancer as it is most frequently diagnosed in women between 35–44 years of age [40]. The use of contraceptives was found to be a significant risk factor for cervical cancer which is in agreement with some previous studies [41,42]. However, age, high fertility, dwelling status (urban or rural), menopausal status, smoking, family history of cancer was not found to be significant risk factors for cervical cancer. This is in contrast with some previous reports where age, post-menopausal bleeding, irregular periods, high fertility were found to be significant risk factors for cervical cancer [43–45]. A previous study with Bangladeshi cervical cancer patients [13] reported that for TP53 codon 72, family history of cancer was a risk factor that we could not find in our present study.

**Table 2:** Distribution of demographic variables of cervical cancer patients & controls.

Characteristics	Cases (n = 129) (%)	Controls (n = 122) (%)	χ^2^ (P value)
***Age*, *years***			
≤ 45	61 (47.3)	69 (56.6)	2.158 (0.165)
> 45	68 (52.7)	53 (43.4)	
***Dwelling***			
Urban	26 (20.2)	21 (17.2)	0.357 (0.628)
Rural	103 (79.8)	101 (82.8)	
***Menstrual Status***			
Pre menopause	74 (57.4)	59 (48.4)	2.040 (0.166)
Post menopause	55 (42.6)	63 (51.6)	
***Tobacco use***			
Smokers	31	20	2.259 (0.133)
Non-smokers	98	102	
***Parity***			
0–7	125 (96.9)	116 (95.1)	0.541 (0.531)
> 7	4 (3.1)	6 (4.9)	
***Contraception***			
Oral Pills	56 (43.4)	67 (54.9)	0.375
Others^§^	15 (11.6)	4 (3.3)	**0.029** *
Combination^#^	10 (7.8)	6 (4.9)	0.420
None	48 (37.2)	45 (36.9)	-
***Family History of Cancer (First Degree Relatives)***			
Yes	21 (16.28)	25 (13.67)	0.389
No	108 (83.72)	97 (86.33)	
***Stage of Cancer (FIGO)***			
IA-IB	25 (19.4)		
IIA-IIB	68 (52.7)		
IIIA-IIIB	36 (27.9)		
***Histopathology***			
Squamous Cell Carcinoma	105 (81.4)		
Adenocarcinoma	18 (14.0)		
Others	6 (4.7)		
***Tumor Grade***			
I	12 (9.3)		
II	101 (78.3)		
III	16 (12.4)		

*P<0.05.

^§^Others: Intrauterine device (IUD) + Barrier (cervical cap, diaphragm, female condom).

^#^Combination: Oral pills + condom (male), Oral pills + combined injectable contraceptives (CIC).

### TP53 codon 72 and codon 47 polymorphisms

Table 3 contains the genotype frequencies for both the SNPs in cases and controls. For codon 72, the distribution of the Pro/Pro mutant homozygous genotype was higher in cases compared to healthy controls (21.7% vs 9%). It contains 3.564 folds more risk of developing cervical cancer with a highly significant P value (adjusted OR = 3.564, 95% CI = 1.647–7.713, P = 0.001). The Arg/Pro heterozygous genotype also confers 2.126 times more odds of developing cervical cancer with a marginally significant P value (adjusted OR = 2.126, 95% CI = 1.181–3.827, P = 0.012). The combined frequency of Arg/Pro and Pro/Pro was also found to be significantly associated with the development of cervical cancer (adjusted OR = 2.542, 95% CI = 1.517–4.260, P<0.001). Association of this SNP with different clinicopathological characteristics in the patient group did not yield any significant result (Table 4). For the codon 47 polymorphism, none of the Pro/Ser and Ser/Ser polymorphisms were significantly associated with the risk of developing cervical cancer.

**Table 3:** Genotype frequencies of *TP53* gene polymorphisms in cervical cancer patients and controls.

	Genotypes	Cases	Controls	Adjusted Odds Ratio (AORs)	95% CI	*P value*
**TP53 codon 47**		**n = 129 (%)**	**n = 122 (%)**			
	Pro/Pro	120 (89.1)	116 (95.1)	Ref.	-	-
	Pro/Ser	7 (7.8)	5 (4.1)	1.353	0.418–4.385	0.614
	Ser/Ser	2 (3.1)	1 (0.8)	1.933	0.173–21.612	0.592
	Pro/Ser +Ser/Ser	9 (6.9)	6 (4.9)	1.450	0.500–4.202	0.494
	Ser Allele	11 (8.5)	7 (5.8)	1.519	0.579–3.986	0.396
**TP53 codon 72**						
	Arg/Arg	60 (46.5)	84 (68.9)	Ref.	-	-
	Arg/Pro	41 (31.8)	27 (22.1)	2.126	1.181–3.827	**0.012** *
	Pro/Pro	28 (21.7)	11 (9.0)	3.564	1.647–7.713	**0.001** **
	Arg/Pro +Pro/Pro	69 (53.5)	38 (31.2)	2.542	1.517–4.260	**<0.001** ***
	Pro Allele	97 (67.5)	49 (40.2)	2.772	1.828–4.201	**<0.001** ***

*P <0.05;

**P <0.01,

***P< 0.001.

**Table 4:** Correlation of *TP53* codon 72 polymorphisms with clinicopathological characteristics of the patients.

Characteristics	Codon 72 carrier	Codon 72 non-carrier	OR (95% CI)	*P*-value
	n = 69	n = 60		
***Age (years)***				
> 45	36	32	0.955 (0.477–1.909)	0.895
≤ 45	33	28	Ref.	1.000
***Dwelling status***				
Urban	17	9	1.853 (0.757–4.536)	0.177
Rural	52	51	Ref.	1.000
***Menstrual status***				
Pre-menopause	38	36	1.224 (0.607–2.467)	0.573
Post menopause	31	24	Ref.	1.000
***Tobacco use***				
Smokers	21	10	0.644 (0.266–1.562)	0.331
Non-smokers	75	23	Ref	1.000
***Parity***				
>7	3	1	2.682 (0.272–26.489)	0.399
0–7	66	59	Ref.	1.000
***Contraception***				
Oral Pills	32	24	0.886 (0.408–1.926)	0.761
Others^§^	7	8	1.351 (0.422–4.319)	0.612
Combination^#^	4	6	1.773 (0.443–7.094)	0.418
None	26	22	Ref.	1.000
***Family history of cancer (first degree relatives)***				
Yes	13	8	1.509 (0.579–3.934)	0.400
No	56	52	Ref.	1.000

^§^Others: Intrauterine device (IUD) + Barrier (cervical cap, diaphragm, female condom).

^#^Combination: Oral pills + condom (male), Oral pills + combined injectable contraceptives (CIC).

### Serum p53 protein expression

**Fig 1:**
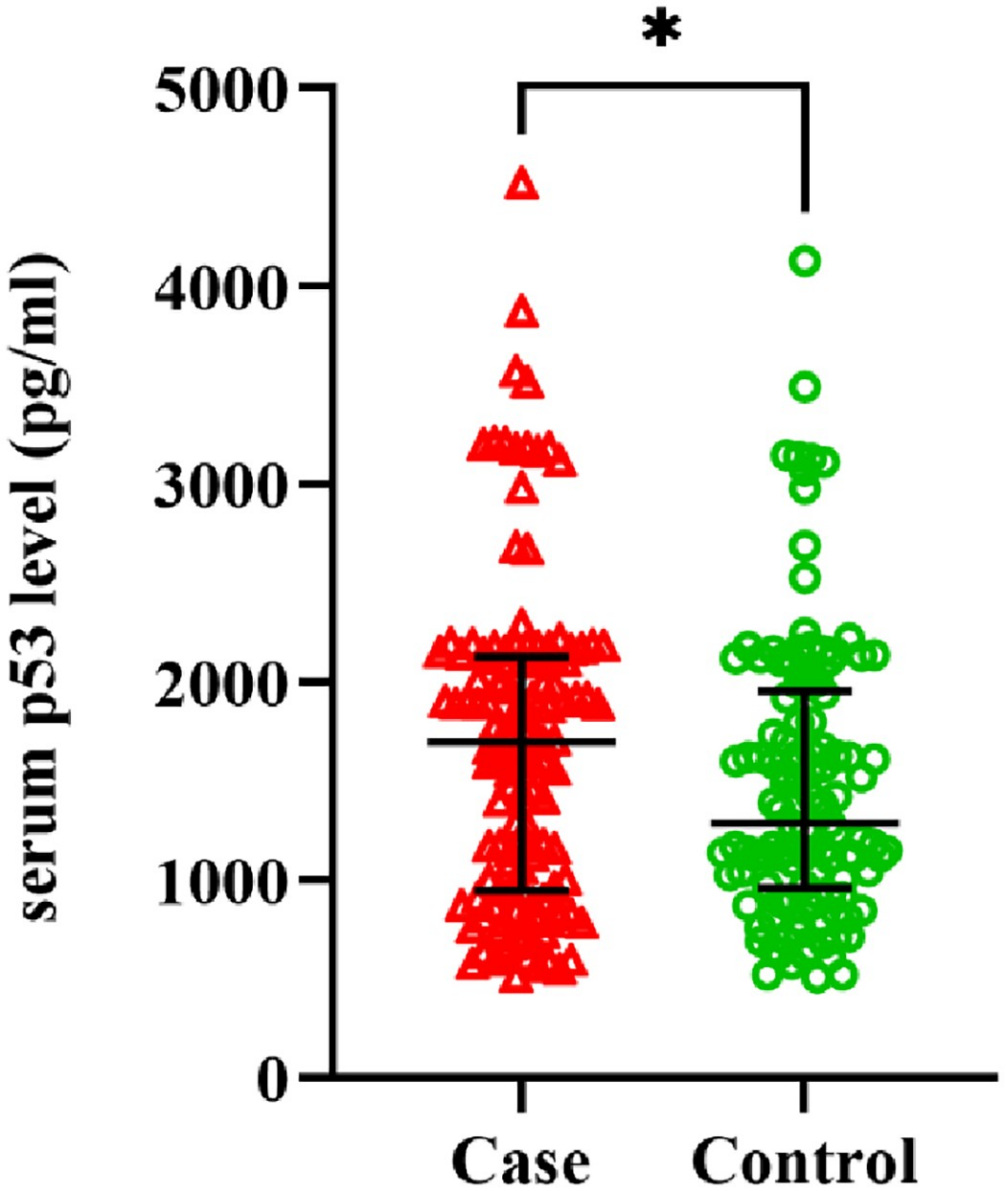
Differences in serum p53 expression level between control and cases with cervical cancer [cases: {median: 1697 pg/ml, IQR: 946–2128}; controls: {median: 1285 pg/ml, interquartile range (IQR): 957.8–1953}; Wald chi-square, (χ^2^) = 3.81, df = 1, adjusted P = 0.0442]. Error bars represent median ± interquartile range *P<0.05.

**Fig 2:**
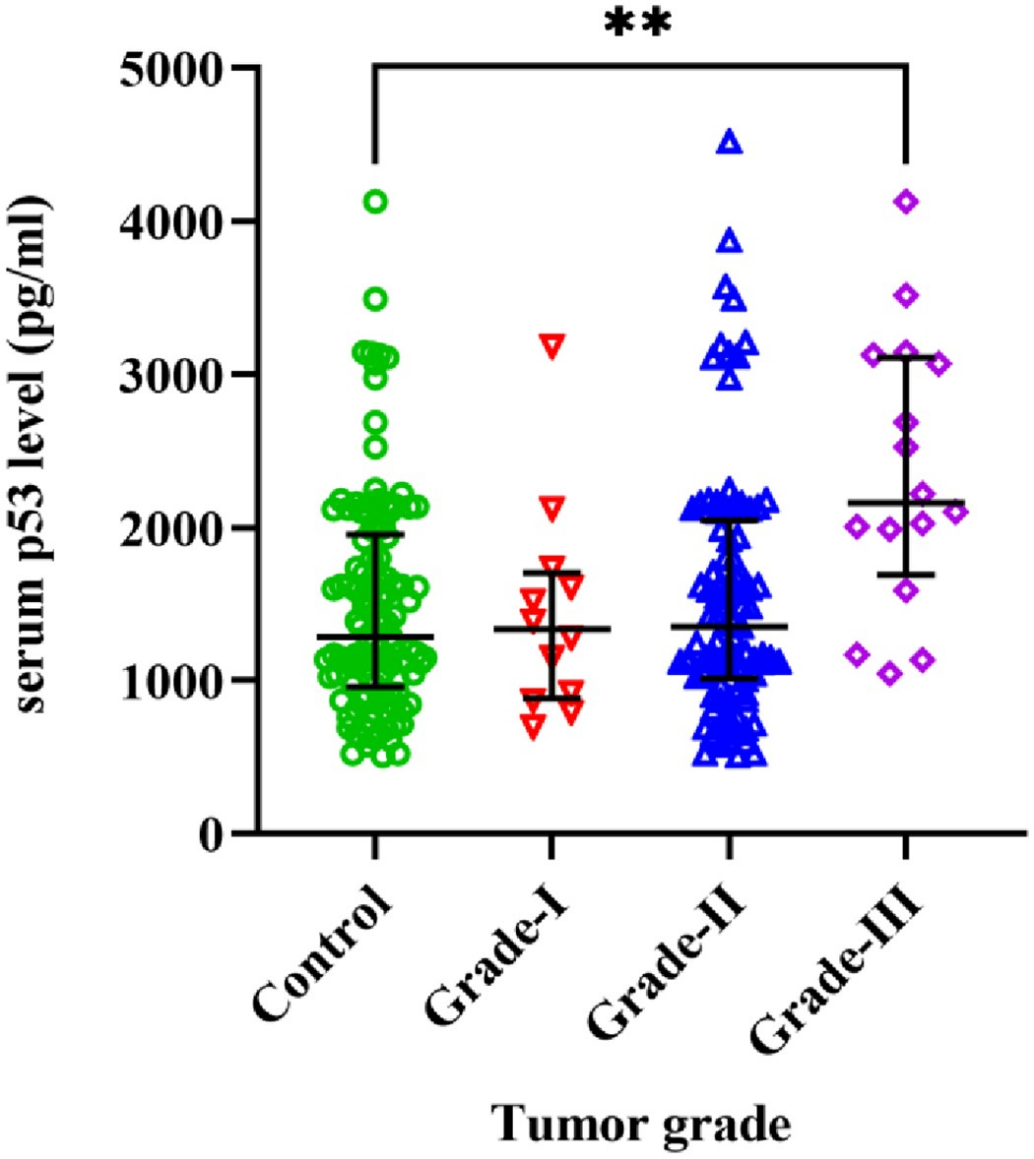
Differences in serum p53 expression between controls vs cases with different grades of tumor (Kruskal-Wallis, H = 11.19, P = 0.004). Level of p53 expression: [(controls: 1285 pg/ml, IQR: 957.8–1953); (cases with grade-I tumor: 1216 pg/ml, IQR: 812.3–1588); (cases with grade-II tumor: 1204 pg/ml, IQR: 921–1796); (cases with grade-III tumor: 2064 pg/ml, IQR: 1275–2974)]. Control vs grade-I tumor (mean rank difference: 2.882, adjusted P>0.99); control vs grade-II (mean rank difference: − 2.836, adjusted P>0.99); control vs grade-III (mean rank difference: -69.13, adjusted P = 0.001). Error bars represent median ± interquartile range. **P<0.01.

Serum p53 protein levels were elevated in cervical cancer patients in comparison to healthy controls (Fig 1). The median p53 level in cases and controls were 1697 pg/ml and 1285 pg/ml respectively and generalized linear model analysis resulted in an adjusted p-value of 0.0442. Correlation analysis of the protein expression with age (Spearman’s rho = -0.019, P = 0.759) and parity (Spearman’s rho = 0.060, P = 0.340) did not show any significant interaction. Kruskal-Wallis test between controls and patients with different grades of the tumor showed that there was a significant interaction (H = 11.19, P = 0.004). Post-hoc multiple comparisons with controls showed that protein level was significantly decreased in patients with grade-III tumor (P = 0.001) (Fig 2). A similar analysis by stratification of the cases according to other histopathological characteristics (stage of cancer, type of cervical cancer) and comparing them with controls did not yield any significant result (S2 Fig).

### Expression quantitative trait loci analysis

Examination of the codon 47 and codon 72 SNPs with protein expression did not reveal any significant result. Using general linear model, for codon 47 the F value was 0.389 (P = 0.678) and for codon 72 the F value was 0.345 (P = 0.709) (S2 Table).

## Discussion

In this study, we found that TP53 codon 72 polymorphism is associated with an increased risk of cervical cancer in the Bangladeshi population. Expression of serum p53 protein is upregulated in patients with cervical cancer. Moreover, we found significantly elevated levels of serum p53 protein in patients with grade-III tumors compared to healthy controls.

TP53 plays a vital role in apoptosis, tumor suppression and polymorphism of the TP53 codon 72 has been associated with increased risk of different types of malignancies including cervical cancer [13,14,16]. In our study, we found that the Arg/Pro heterozygous genotype and Pro/Pro mutant homozygous genotype carry 2.126 and 3.564 more odds of developing cervical cancer respectively. The frequency of the Proline allele was found higher in cases compared to healthy controls (21.7% vs 9%). A previous study by our group in a separate cohort of cervical cancer patients from Bangladesh reported for the first time that the Arg/Pro heterozygous genotype and Pro/Pro mutant homozygous genotype carry an increased risk of cervical cancer in Bangladeshi women [13]. This study is in line with the previous findings for Bangladeshi population. The Proline allele has less apoptosis-inducing ability compared to the Arginine allele as it cannot interact directly with the BAK protein and thus may lead to tumorigenesis [46]. Our finding is also supported by a meta-analysis with Han Chinese women where the Proline allele was reported to be associated with an increased risk of cervical cancer [47]. On the contrary, studies in patients with cervical cancer from Serbia and Saudi Arabia did not report an increased risk of the malignancy with the Proline allele [22,24]. Interestingly, the Proline allele was reported to impart a protective effect on cervical cancer [22]. The role of the Proline allele may be different in women from different ethnicities. Further studies need to be conducted to elucidate the effect of the Proline allele in cervical cancer in women from different ethnic groups. In this study, we did not find any significant transmission of the Proline allele in cervical cancer patients who had first-degree relatives with cancer which we found in our previous study [13]. However, we found a significant difference in the use of contraceptives as women using intrauterine devices and barriers (diaphragm, cervical cap, female condom) had higher rates of cervical cancer in comparison to controls (P = 0.029). The soft silicone and synthetic latex used in these barriers may have some carcinogenic properties which is supported to some extent by previous studies where synthetic rubber industry workers had high incidence of cancer [48,49]. Further studies need to be conducted to find out how synthetic latex and soft silicone may increase the risk of cervical cancer among women using intrauterine devices and barriers as contraceptives. No significant association was found between codon 47 (Pro47Ser) polymorphism with cervical cancer which is also supported by our previous study [13].

The majority of investigation of p53 protein in cervical cancer has been done in tissue biopsies with immunohistochemical staining [36,50,51]. To the best of our knowledge, only a limited number of studies investigated the serum p53 level in cervical cancer. Serum p53 has the potential to be used as a biomarker for different types of cancer [52,53]. Our finding of elevated levels of serum p53 protein in cervical cancer patients is in agreement with a previous study where they reported upregulation of p53 protein in patients with invasive cervical carcinoma [30]. Another study by Barbati et al. reported that serum p53 levels were increased in patients with cervical cancer [34]. Elevated expression of p53 in tissue biopsies has also been reported previously by several studies [54–56], which suggests that peripheral expression may be used as a proxy for malignant tissue expression. In contrast, reports of no significant difference in serum p53 levels between cervical cancer patients and controls have been reported in the Korean population [35]. A recent study in north-east Indian cervical cancer patients reported significant downregulation of p53 mRNA and protein in tissue biopsies compared to non-neoplastic control areas but a majority of the patients were affected with HPV infection [57] where viral oncogene E6 may be responsible for the degradation of the p53 protein [58]. So, p53 may be used as a peripheral biomarker only in cervical cancer patients without HPV infection. We found no difference for serum p53 expression in groups with different types of cancer or different cancer stage which suggests that peripheral p53 expression alone may have a poor prognostic value for cervical cancer which has been hypothesized by previous studies [59,60]. However, we found upregulation of serum p53 in patients with grade-III tumor vs controls only but no significant difference between patients with different grades of tumor (Fig 2). This leads to the idea that elevated peripheral expression of p53 may be a characteristic only in patients with high-grade tumors as the serum levels of p53 may fluctuate due to tumor lysis and reduced clearance of p53 protein from blood [30]. Further studies need to be conducted to find out the potential of using peripheral expression of p53 as a biomarker in cervical cancer.

In addition, we found no cis-regulatory effect of codon 72 and codon 47 on serum p53 protein expression. Genotyping of more SNPs is warranted to find out the putative polymorphisms that increase the risk of cervical cancer and act as eQTL for tissue and peripheral biomarkers.

Several limitations must be noted. Our sample size was relatively small and only allowed for detection of moderate to large differences in protein expression and may lack enough power to detect genotype-disease association. Secondly, the cross-sectional design of the study does not allow detection of the pattern of changes of serum p53 expression with time and how they relate to clinical outcomes. Thirdly, the generalizability of our findings to other peripheral tissues (plasma, lymphocytes) and tumor tissues is not clear.

Future studies with larger sample size and different ethnicities are warranted to elucidate whether mutation of TP53 codon 72 increases the risk of cervical cancer or gives a protective effect. Moreover, the difference in expression of different isoforms of TP53 in mRNA and protein level needs to be investigated to find out the suitable biomarker for clinical use. More SNPs need to be genotyped to find out suitable expression quantitative trait loci for mRNA and protein expression in cancer tissue and in the periphery.

Despite this, we report for the first time the upregulation of serum p53 protein in patients with cervical cancer from the Bangladeshi population. Moreover, we found a significant upregulation of serum p53 protein in patients in high-grade tumors. We also provide more evidence on the association of TP53 codon 72 polymorphisms with increased risk of cervical cancer in Bangladeshi women.

## Supporting information

S1 Fig Normal Q-Q plots for serum p53 protein expression. (DOCX)

S2 Fig Differences in serum p53 protein expression between controls and cases with different histopathological characteristics. (DOCX)

S1 Table Details of the PCR–RFLP experiment. (DOCX)

S2 Table Expression quantitative trait loci analysis. (DOCX)
