# Supplementary material for: Elevated serum expression of p53 and association of TP53 codon 72 polymorphisms with risk of cervical cancer in Bangladeshi women
Source: PLoS One. 2021 Dec 28;16(12):e0261984. doi: 10.1371/journal.pone.0261984 (PMC8714093; doi:10.1371/journal.pone.0261984)
Supplement: S1 Fig — (DOCX) [file pone.0261984.s001.docx]

**Supplementary Figure S1**


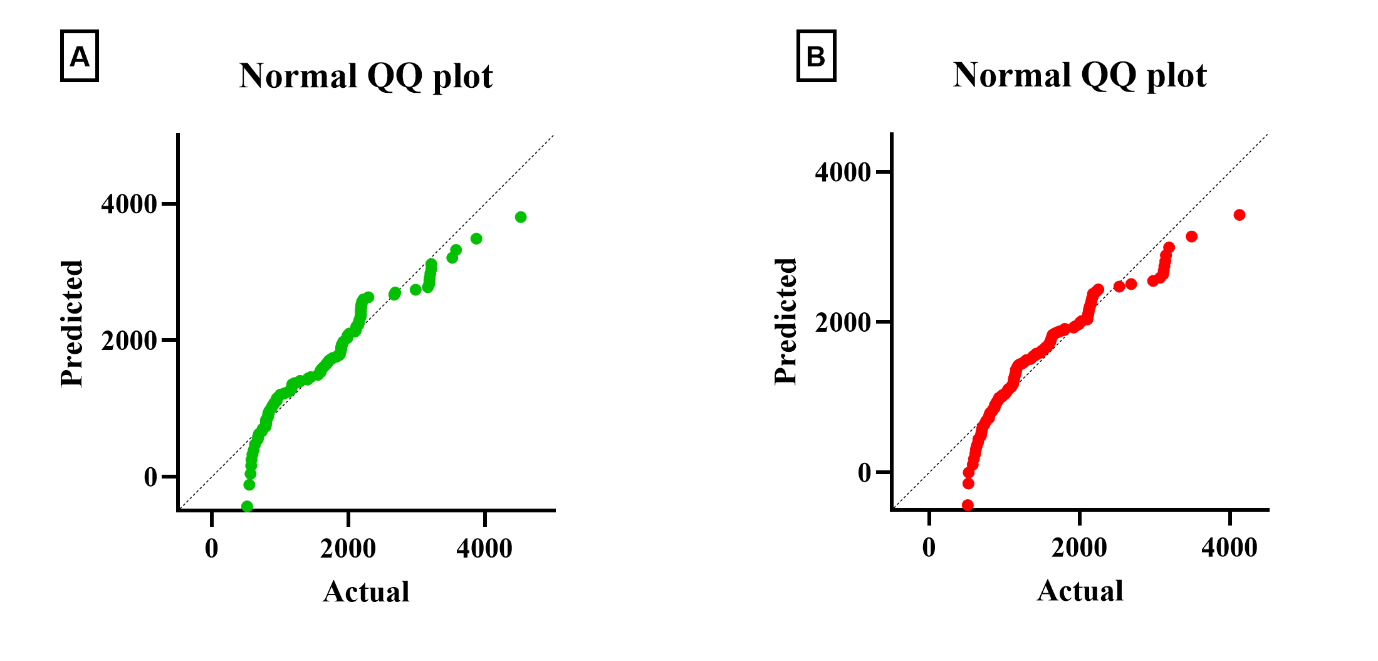


**Supplementary Figure S1:** Normal Q-Q plots for serum p53 protein expression, A. Normal Q-Q plot for controls (Shapiro-Wilk, W=0.9325, P<0.0001); B. Normal Q-Q plot for cervical cancer cases (Shapiro-Wilk, W=0.9124, P<0.0001).
