# Supplementary material for: Elevated serum expression of p53 and association of TP53 codon 72 polymorphisms with risk of cervical cancer in Bangladeshi women
Source: PLoS One. 2021 Dec 28;16(12):e0261984. doi: 10.1371/journal.pone.0261984 (PMC8714093; doi:10.1371/journal.pone.0261984)
Supplement: S2 Fig — (DOCX) [file pone.0261984.s002.docx]

**Supplementary Figure S2**


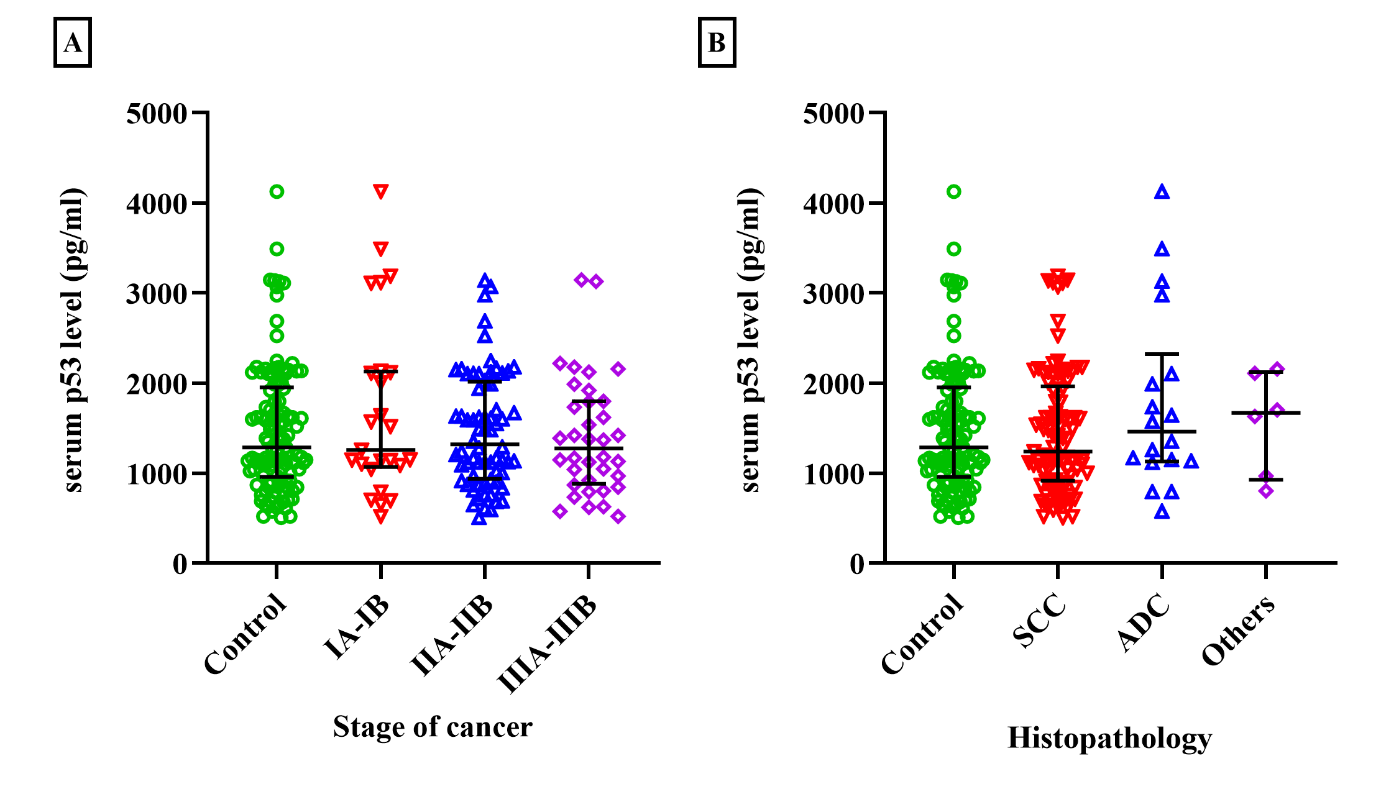


**Supplementary Figure S2:** Differences in serum p53 protein expression between controls and cases with different histopathological characteristics; A. Controls vs cases with different stages of cervical cancer (Kruskal-Wallis, H=0.7431, P=0.8630); B. Controls vs cases with different types of cervical cancer (Kruskal-Wallis, H=1.793, P=0.6164).

SCC= squamous cell carcinoma

ADC= adenocarcinoma
