# Supplementary material for: Elevated serum expression of p53 and association of TP53 codon 72 polymorphisms with risk of cervical cancer in Bangladeshi women
Source: PLoS One. 2021 Dec 28;16(12):e0261984. doi: 10.1371/journal.pone.0261984 (PMC8714093; doi:10.1371/journal.pone.0261984)
Supplement: S1 Table — (DOCX) [file pone.0261984.s003.docx]

**Supplementary Table S1:** Details of the PCR–RFLP experiment

| **SNPs** | **Primer** | **PCR condition** | **PCR product** | **Restriction enzyme** | **PCR fragment** |
| --- | --- | --- | --- | --- | --- |
| rs1800371  (Codon 47  Pro47Ser)  C>T | Forward: 5′-CTG GTA AGG ACA AGG GTT GG-3′  Reverse: 5 ′-TCA TCT GGA CCT GGG TCT  TC-3′ | 94 for 5 min  94 for 30 s  54 for 30 s  72 for 30 s  72 for 5 min  35 cycles | 201  or  185 | *Msp*I  (1U at 37 °C for 16 h) | NH=156/140, 45  HE=201/185, 156/140, 45  MH=201/185 |
| rs1042522  (Codon 72  Arg72Pro)  G>C | Forward: 5′-TTC ACC CAT CTA CAG TCC-3′  Reverse: 5′-CTC AGG GCA ACT GAC CGT-3 | 94 for 5 min  94 for 30 s  54 for 30 s  72 for 30 s  72 for 5 min  35 cycles | 309 | *BstU*I  (1U at 37 °C for 16 h) | NH=175, 134  HE=309, 175, 134  MH=309 |

NH= normal homozygote, HE=heterozygote, MH=mutant homozygote, RE=restriction enzyme.
