# Supplementary material for: Elevated serum expression of p53 and association of TP53 codon 72 polymorphisms with risk of cervical cancer in Bangladeshi women
Source: PLoS One. 2021 Dec 28;16(12):e0261984. doi: 10.1371/journal.pone.0261984 (PMC8714093; doi:10.1371/journal.pone.0261984)
Supplement: S2 Table — (DOCX) [file pone.0261984.s004.docx]

**Supplementary Table S2:** Expression quantitative trait loci analysis

| ***TP53* SNPs** | **p53 protein expression**  **F value (P)** |
| --- | --- |
| Codon 47 | 0.389 (0.678) |
| Codon 72 | 0.345 (0.709) |
